# RIG-I expression in perifascicular myofibers is a reliable biomarker of dermatomyositis

**DOI:** 10.1186/s13075-017-1383-0

**Published:** 2017-07-24

**Authors:** Xavier Suárez-Calvet, Eduard Gallardo, Iago Pinal-Fernandez, Noemi De Luna, Cinta Lleixà, Jordi Díaz-Manera, Ricardo Rojas-García, Ivan Castellví, M. Angeles Martínez, Josep M. Grau, Albert Selva-O’Callaghan, Isabel Illa

**Affiliations:** 1 Neuromuscular Diseases Unit, Neurology Department, Hospital de la Santa Creu i Sant Pau and Institut de Recerca Sant Pau, Universitat Autònoma de Barcelona, Sant Antoni Maria Claret 167, 08025 Barcelona, Spain; 2 Center for Biomedical Network Research on Rare Diseases (CIBERER), Madrid, Spain; 3 Autoimmune Systemic Diseases Unit, Department of Internal Medicine, Hospital Vall d’Hebron, Universitat Autònoma de Barcelona, Barcelona, Spain; 4 Rheumatology Unit, Hospital de la Santa Creu i Sant Pau, Barcelona, Spain; 5 Immunology Department, Hospital de La Santa Creu I Sant Pau, Universitat Autonoma de Barcelona, Barcelona, Spain; 6 Muscle Research Unit, Internal Medicine Service, Hospital Clínic de Barcelona, Barcelona, Spain

**Keywords:** Inflammatory myopathies, Dermatomyositis, Biomarker, Muscle biopsy, Perifascicular atrophy

## Abstract

**Background:**

Dermatomyositis (DM) is inflammatory myopathy or myositis characterized by muscle weakness and skin manifestations. In the differential diagnosis of DM the evaluation of the muscle biopsy is of importance among other parameters. Perifascicular atrophy in the muscle biopsy is considered a hallmark of DM. However, perifascicular atrophy is not observed in all patients with DM and, conversely, perifascicular atrophy can be observed in other myositis such as antisynthetase syndrome (ASS), complicating DM diagnosis. Retinoic acid inducible-gene I (RIG-I), a receptor of innate immunity that promotes type I interferon, was observed in perifascicular areas in DM. We compared the value of RIG-I expression with perifascicular atrophy as a biomarker of DM.

**Methods:**

We studied by immunohistochemical analysis the expression of RIG-I and the presence of perifascicular atrophy in 115 coded muscle biopsies: 44 patients with DM, 18 with myositis with overlap, 8 with ASS, 27 with non-DM inflammatory myopathy (16 with polymyositis, 6 with inclusion body myositis, 5 with immune-mediated necrotizing myopathy), 8 with muscular dystrophy (4 with dysferlinopathy, 4 with fascioscapulohumeral muscle dystrophy) and 10 healthy controls.

**Results:**

We found RIG-I-positive fibers in 50% of DM samples vs 11% in non-DM samples (*p* < 0.001). Interestingly, RIG-I staining identified 32% of DM patients without perifascicular atrophy (*p* = 0.007). RIG-I sensitivity was higher than perifascicular atrophy (*p* < 0.001). No differences in specificity between perifascicular atrophy and RIG-I staining were found (92% vs 88%). RIG-I staining was more reproducible than perifascicular atrophy (κ coefficient 0.52 vs 0.37).

**Conclusions:**

The perifascicular pattern of RIG-I expression supports the diagnosis of DM. Of importance for clinical and therapeutic studies, the inclusion of RIG-I in the routine pathological staining of samples in inflammatory myopathy will allow us to gather more homogeneous subgroups of patients in terms of immunopathogenesis.

**Electronic supplementary material:**

The online version of this article (doi:10.1186/s13075-017-1383-0) contains supplementary material, which is available to authorized users.

## Background

Dermatomyositis (DM) is an inflammatory myopathy characterized by proximal muscle weakness, the presence of perifascicular atrophy (PFA) in the muscle biopsy and skin changes. Muscle biopsy also shows overexpression of the major histocompatibility complex class I (MHC-I) in the muscle fibers that is more prominent in the atrophic perifascicular areas [1]. It has been reported that a high percentage of patients with DM do not show PFA in the muscle biopsy [2, 3], and conversely, patients with antisynthetase-associated myopathy may have PFA [4, 5]. Moreover, to our knowledge the presence of PFA in the group of overlap myositis that is included in the classification of adult autoimmune myositis [6] has not been explored. All these findings suggest that the diagnosis of DM based on the presence of PFA should be made with caution.

The role of interferons (IFN) and innate immunity in DM is an emerging field of research [7, 8]. Type I IFN mechanisms have been associated with DM pathogenesis [8] and some IFN-I-induced proteins such as MxA have been shown to be present in perifascicular muscle fibers in DM [8]. It has been recently reported that the evaluation of MxA expression in the muscle biopsy is a biomarker for DM [5]. We previously reported the overexpression of another IFN-I-induced protein called retinoic acid inducible-gene I (RIG-I, the product of the *DDX58* gene) in perifascicular areas in DM biopsies in five out of five patients [9]. RIG-I is a member of the RLR family of innate immune receptors that recognize dsRNA and 5′-triphosphate ssRNA [10]. In addition, our in vitro studies using human skeletal muscle primary cultures have shown the ability of RIG-I to induce type I IFN responses and in turn upregulates MHC-I and RIG-I itself [9], suggesting a specific self-sustained pathogenic mechanism independent of inflammatory infiltrates [11]. We aimed to determine if RIG-I staining is a reliable diagnostic marker of DM compared with PFA in neuromuscular diseases to validate its sensitivity and specificity using a large cohort of patients.

## Methods

### Patients

We collected 115 muscle biopsies performed for diagnostic purposes from three hospitals in Barcelona (Sant Pau, Vall d’Hebron, Clínic). Routine histochemical analyses were performed. We included 44 patients with definite DM (without criteria for any other of the clinical [4, 12] situations mentioned subsequently), 16 with polymyositis (PM), 6 with inclusion body myositis (IBM), 5 with immune-mediated necrotizing myopathy (IMNM), 4 with dysferlinopathy (Dysf), 4 with fascioscapulohumeral muscle dystrophy (FSHD), 18 patients with various autoimmune systemic diseases (rheumatoid arthritis (RA), systemic lupus erythematosus (SLE), mixed connective tissue disease (MCTD), systemic sclerosis (SSc) and Sjögren syndrome (SS)) with overlapped myopathy (OM) and 8 with antisynthetase syndrome (ASS), who were not included in the DM group due to recent studies that indicate its distinctiveness among the inflammatory myopathies [4, 12]. Biopsies were a representative subset of the biopsy collection that we have in each of our centers and were not selected based on any specific clinical or histological feature. As healthy controls we included 10 muscle samples from subjects undergoing orthopedic surgery. Routine histological stains were normal. Healthy control samples were not included in the statistical analysis.

The diagnosis of DM, PM, IBM and IMMN was based on the ENMC criteria [13]. The diagnosis of each autoimmune systemic disease was based on established criteria [14]. Dysf and FSHD diagnosis were confirmed by genetic analysis.

### Serum autoantibodies

Anti-TIF1γ and anti-MDA5 were detected by an in-house enzyme-linked immunosorbent assay (ELISA) and confirmed by immunoblot [15, 16]. Other synthetases and myositis-specific and myositis-associated antibodies were tested using a commercial kit for myositis-antigen profile (Euroimmun, Luebeck, Germany): anti-Mi-2, anti-SRP, anti-Jo1, anti-Ku, anti-PM/Scl, anti-PL7, anti-PL12, anti-EJ, anti-OJ and anti-Ro.

### Immunohistochemical analysis

Immunohistochemical analysis of RIG-I and MHC-I was performed on coded samples and the slides were read blinded by six investigators (XSC, EG, IPF, JMG, ASO and II). Serial sections were incubated with 3% hydrogen peroxide (Novocastra Peroxidase Block, Novocastra Leica Microsystems) to neutralize endogenous peroxidase activity, fixed in acetone for 5 min, washed with Tris-buffered saline (TBS) and incubated with Novocastra Protein Block (Novocastra). Sections were then incubated with anti-RIG-I (Thermo Scientific, Rockford, USA) at 1/50 or with anti-MHC-I (Dako, Carpenteria, CA, USA) at 1/100 or with anti-MxA at 1/50 (Santa Cruz laboratories, Dallas, TX, USA) O/N at 4 °C. After washing steps, sections were treated with Novocastra Postprimary Block containing 10% (v/v) animal serum in TBS. Poly-horseradish-peroxidase (HRP) anti-mouse/rabbit IgG reagent (NovoLink Polymer) containing 10% (v/v) animal serum in TBS was applied to localize the primary antibody. The reaction product was visualized by incubation with the substrate/chromogen, 3,3′-diaminobenzidine (DAB) prepared from Novocastra DAB Chromogen and NovoLink DAB Substrate Buffer (Polymer), as a brown precipitate, and mounted with aquatex. Images were obtained using an Olympus BX51 microscope coupled to a DP72 camera. Slides were considered as positive when RIG-I expression was observed in at least one row of perifascicular muscle fibers. To confirm the specificity of RIG-I antibody, we used HEK293 cells transfected with a RIG-I constitutive expression vector (pCMV-RIG-I; Origene) using Fugene HD Transfection system (Promega). Non-transfected cells were used as a negative control (Additional file 1: Figure S1).

### Analysis of perifascicular atrophy

There are no established criteria to evaluate PFA. We performed a morphometric study measuring the minimum Feret’s diameter in the muscle biopsies from patients with DM (n = 4), PM (n = 4), MCTD (n = 4) and from controls (n = 3), stained with anti-MHC-I (Additional file 2: Figure S2). We compared these objective results with those of three independent observers asked to assess the presence of atrophy and we obtained similar results. Muscle biopsies with PFA were considered positive when one or more rows of perifascicular muscle fibers had a reduction in diameter. We also analyzed the presence of PFA using a more restrictive criterion that included only those biopsies with two or more rows of atrophic fibers. Therefore, the second group is included in the first group. Both groups were analyzed separately. Muscle biopsies were read blinded by five independent investigators (XSC, EG, IPF, II and NL).

### Statistics

Fisher’s exact test or the chi square (χ^2^) test if appropriate was used to compare RIG-I positivity between patients with and without DM. The reproducibility of the biopsy results between observers was assessed using Cohen’s kappa coefficient (κ), considering a coefficient over 0.75 as excellent, between 0.4 and 0.75 as fair and below 0.4 as poor. Sensitivity and specificity of RIG-I and PFA to detect DM were compared using the McNemar test. Statistical analyses were performed using Stata v.13 software. A *p* value <0.05 was considered significant.

## Results

### Clinical data

All patients with myopathy included in the study had muscle weakness evaluated by the Medical Research Council (MRC) scale. Antibodies were tested using the Euroline system in 41 patients with DM, 12 with PM, 6 with IBM, 5 with IMNM, 8 with ASS and 18 with OM. Anti-ARS antibodies were only found in eight patients with ASS and in one patient with OM. The antibody profiling showed the presence of anti-Ku in 2/8 patients with OM, while all patients from the other groups tested negative (41 with DM and 12 with PM). Reactivity against anti-PM/Scl (2/26), anti-Jo-1 (9/26) and anti-Ro52 (6/26) were only found in patients with ASS and OM. Anti-Mi-2 (3/41), anti-MDA5 (1/41) and anti-TIF1g (2/41) were only positive in patients with DM. Anti-SRP antibodies were only found in one patient with IMNM. Anti-EJ, anti-OJ, anti-PL7 and anti-PL12 were negative in all the patients tested.

### Perifascicular atrophy affecting two rows of muscle fibers is more frequently associated with DM

We found that 28% of the pathological muscle biopsies included had perifascicular muscle fibers with a reduction in fiber diameter. In contrast, we found two or more rows of atrophic fibers, a more restrictive criterion, in only 15% of all pathology samples (Table 1). When we analyzed patients with DM, we found that 36% of DM samples (16/44) displayed PFA that was restricted to 25% (11/44) when we considered two or more rows (Table 1). In non-DM samples we found that 21% (13/61) had PFA (7 ASS, 3 SSc, 1 Sjögren, 1 MCTD and 1 SLE sample). When we considered two or more rows, the percentage decreased to 8% (5/61) (1 ASS, 2 SSc, 1 MCTD and 1 RA sample). Therefore, ASS samples were significantly associated with a single row of atrophic fibers (*p* < 0.001) (Table 1). Statistical analysis showed that PFA was not significantly associated with DM (*p* = 0.089), although it was suggestive of DM. In contrast, PFA affecting two or more rows was significantly associated with DM (*p* = 0.018) (Table 2).

**Table 1:** Summary of the results

	Diagnosis	Perifascicular atrophy^a^	Perifascicular atrophy (2 rows)	RIG-I+	Total
Inflammatory myopathy (n = 71)	DM	16 (36%)	11 (25%)	22 (50%)	44
	PM	0 (0%)	0 (0%)	0 (0%)	16
	IBM	0 (0%)	0 (0%)	2 (33%)	6
	IMNM	0 (0%)	0 (0%)	0 (0%)	5
Overlapped myopathy (n = 26)	ASS^b^	7 (88%)	1 (13%)	1 (13%)	8
	RA	0 (0%)	0 (0%)	1 (50%)	2
	SLE	1 (50%)	1 (50%)	1 (50%)	2
	SSc	3 (60%)	2 (40%)	1 (20%)	5
	Sjögren	1 (20%)	0 (0%)	0 (0%)	5
	MCTD	1 (25%)	1 (25%)	1 (25%)	4
Muscle dystrophy (n = 8)	Dysf	0 (0%)	0 (0%)	0 (0%)	4
	FSHD	0 (0%)	0 (0%)	0 (0%)	4
Total		29 (28%)	16 (15%)	29 (28%)	105

Healthy controls were excluded from this analysis. *DM* dermatomyositis, *PM* polymyositis, *IBM* inclusion body myositis, *IMNM* immune-mediated necrotizing myopathy, *ASS* anti-synthetase syndrome, *RA* rheumatoid arthritis, *SLE* systemic lupus erythematosus, *SSc* scleroderma and scleromyositis, *MCTD* mixed connective tissue disease, *Dysf* dysferlinopathy, *FSHD* fascioscapulohumeral muscle dystrophy. ^a^Perifasicular atrophy includes one or more rows of atrophic muscle fibers. ^b^The group of ASS patients was established by the presence of anti-Jo-1 antibodies

**Table 2:** Presence of perifascicular atrophy and RIG-I staining in the muscle biopsies of patients with DM and non-DM (1) and sensitivity and specificity of atrophy and RIG-I positivity to diagnose DM (2)

1)						
	PFA^#, a^	PFA (2 rows)^b^	RIG-I^c^			
DM (N; %)	16 (36%)	11 (25%)	22 (50%)			
No DM (N; %)	13 (21%)	5 (8%)	7 (11%)			
p*	0.09	0.02	<0.001			
2)						
				*p* (a vs b)^**^	*p* (a vs c)^**^	*p* (b vs c)^**^
Sensitivity	36%	25%	50%	0.06	0.08	0.005
Specificity	79%	92%	88%	<0.01	0.08	0.7
Reproducibility (κ)^***^		0.37	0.51			

Healthy controls were excluded from this analysis. *DM* dermatomyositis, *PFA* perifascicular atrophy. ^#^Perifascicular atrophy includes one or more rows of atrophic muscle fibers. ^*^Association between bivariate variables was perfomed using Fisher’s exact test. ^**^Sensitivity and specificity were compared using the McNemar test. ^***^Reproducibility was assessed using Cohen’s kappa coefficient (κ).

### Perifascicular RIG-I expression is associated with DM

**Fig. 1:**
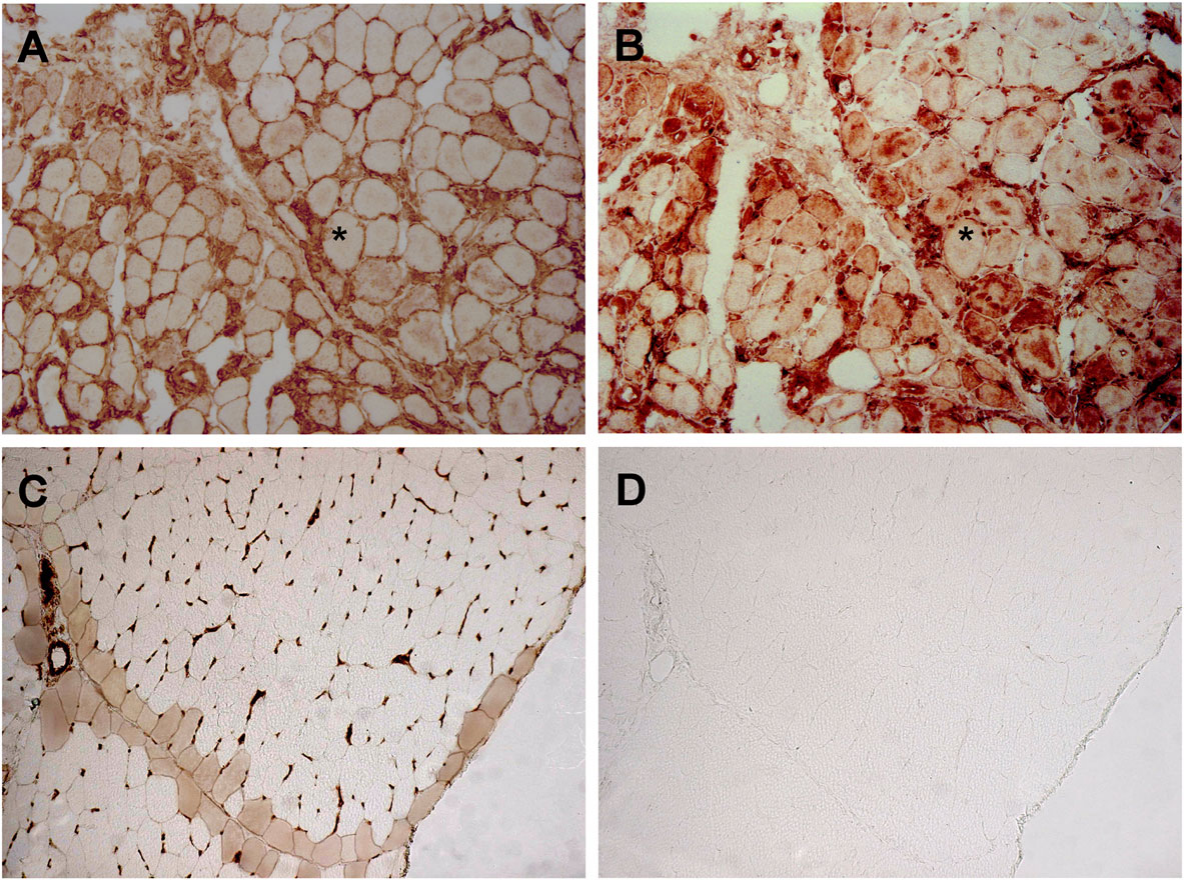
Representative major histocompatibility complex I (MHC-I) and RIG-I staining in serial sections of muscle biopsies. **a** MHC-I is expressed in the muscle fibers from a patient with dermatomyositis (DM) displaying perifascicular atrophy. **b** RIG-I is expressed in perifascicular areas in the sample from a patient with DM. **c** MHC-I is expressed in perifascicular atrophic areas in the sample from a patient with mixed connective tissue disease, whereas RIG-I is not expressed (**d**). Original magnification × 100

MHC-I overexpression was found in all DM samples as expected (Fig. 1a) and RIG-I expression in perifascicular areas was found with a higher frequency in DM samples (Fig. 1b) compared to the other pathology samples. RIG-I perifascicular expression was observed in 50% (22/44) of DM samples but not in the majority of non-DM samples (Table 1). We only found RIG-I expression in 11% (7/61) of non-DM samples: 1 SLE and 1 MCTD sample (both with two or more rows of PFA) and in 1 ASS, 2 IBM, 1 RA and 1 SSc sample (none of them with PFA). Statistical analysis demonstrated that RIG-I is significantly associated with DM (*p* < 0.001) (Table 2). MHC-I overexpression was also found in non-DM samples and in some cases, it was restricted to one row of perifascicular atrophic fibers (Fig. 1c) but RIG-I was negative (Fig. 1d), indicating the usefulness of RIG-I staining.

### Value of combined analysis of perifascicular atrophy and RIG-I for the diagnosis of DM

We compared the utility of PFA (one or two or more rows of muscle fibers) and RIG-I staining for differential diagnostic purposes. The specificity of PFA for DM diagnosis based on two rows is significantly higher than considering one row of muscle fibers (92% vs 79%: *p* < 0.01) (Table 2). We did not find significant differences in sensitivity comparing PFA in general vs PFA restricted to two or more rows, although we observed a clear tendency ﻿toward better sensitivity for PFA in general﻿ (36% vs 25%; *p* = 0.06) (Table 2). Perifascicular RIG-I expression showed significantly higher sensitivity compared to the presence of two rows of atrophic perifascicular muscle fibers (50% vs 25%; *p* < 0.01), as three non-DM samples (two SSc and one ASS sample) displayed two or more rows of atrophy but were RIG-I-negative. In contrast, we did not find significant differences in sensitivity comparing RIG-I with PFA in general (50% vs 36%) (*p* = 0.08). We did not find significant differences in specificity comparing RIG-I with PFA (one or more rows) (*p* = 0.08) and two rows of PFA (*p* = 0.72) (Table 2). Interestingly, in the absence of PFA, 32% of DM samples (9/28) had perifascicular RIG-I-positive staining, while in non-DM samples RIG-I was observed in only 6% (3/48) (*p* = 0.007). We also found significant differences in RIG-I between DM and OM (50% vs 19%; *p* = 0.01). We compared the reproducibility between the interpretation of RIG-I staining and PFA. We found that RIG-I staining was more reproducible than PFA (κ coefficient 0.51 vs 0.37). In summary, for DM diagnosis the higher sensitivity was obtained with RIG-I expression (50%) and the higher specificity was obtained with two or more rows of perifascicular atrophic muscle fibers (Table 2).

### Comparison of RIG-I and MxA staining

It has been shown that MxA staining in DM biopsies can be useful as a biomarker. We analyzed MxA expression using immunohistochemical assessment in 23 muscle biopsies from our cohort (10 patients with DM, 3 with IBM, 3 with PM, 4 with OM and 3 healthy controls), including patients with DM who were negative for RIG-I, to see if the combination of both biomarkers may improve the diagnostic power. Among the 10 patients with DM who we analyzed, 50% were negative for both RIG-I and MxA and did not show PFA. Two biopsies were double-positive, one with PFA and the other without PFA. Two biopsies were single-positive for RIG-I, one with PFA and the other without PFA, and one biopsy was single-positive for MxA, displaying PFA (Additional file 3: Figure S3). All the patients without DM and healthy controls were double-negative.).

## Discussion

We showed that perifascicular RIG-I expression in the muscle biopsy is a good biomarker for the diagnosis of patients with DM. We based our conclusions on the following results: (1) PFA is not restricted to DM muscle biopsies, (2) the evaluation of PFA does not always differentiate DM from non-DM samples, (3) the evaluation of PFA affecting two or more rows of fibers differentiates DM from non-DM but with low sensitivity and low reproducibility and (4) RIG-I has higher sensitivity, equivalent specificity and higher reproducibility than PFA of two or more rows of fibers. Therefore, the inclusion of RIG-I in the panel of markers to evaluate the muscle biopsy can also be informative in ruling out the diagnosis of DM in muscle biopsies from patients with overlap syndromes showing PFA.

Perifascicular RIG-I expression was found in 50% of patients with DM and in only 11% of patients with non-DM, demonstrating that this marker is significantly associated with DM (*p* < 0.001). In addition, RIG-I staining had significantly higher sensitivity for DM than considering two or more rows of perifascicular atrophic fibers. We found that 32% of DM samples that did not show PFA were RIG-I positive. This result supports the utility of RIG-I staining for the pathological diagnosis of DM. We also found higher reproducibility for RIG-I staining than for PFA. We believe that these differences in reproducibility are in part due to the fact that RIG-I staining is more easily interpretable than PFA.

PFA is considered a hallmark of DM although it is not found in all patients with DM and is not restricted to DM, since it may be also present in other entities such as overlap myositis [2] or ASS [4]. The specificity of PFA was significantly higher when considering two or more rows compared to one row of perifascicular atrophic fibers (92% vs 79%). These results are probably due to the fact that most of the samples displaying a single row of PFA corresponded to ASS. In a study by Troyanov et al. the authors reported that PFA can be seen as frequently in overlap myositis DM (30%) as in pure DM (17%) [2]. In contrast, in another study the authors found that the percentage of PFA in DM can reach 51% [3]. Since the frequency of PFA in DM can be variable, the need for other pathological markers for DM is of interest. Uruha et al. recently reported the evaluation of the IFN-I-induced protein MxA for DM diagnosis [5]. They found that MxA is a valuable biomarker of DM with high sensitivity and specificity. There are other proteins that are induced by IFN-I. As the authors stated in their study, it is necessary to evaluate other IFN-I-induced proteins, such as RIG-I, which has been analyzed in the present study, to find the one that is more sensitive, or the use of a combination of different markers. In fact, our study in a smaller group of patients showed that the combination of both markers detected patients with DM more efficiently. Although both MxA and RIG-I display good value as biomarkers of DM, we have also evaluated overlapped myopathies in addition to ASS, which may present with PFA and therefore they may be included in the differential diagnosis of DM. We indeed found that RIG-I staining is also useful to discriminate between definite DM and overlapped myopathies. Along the same lines, it has been reported that the distinction between the different subtypes of inflammatory myopathies and other entities that may share similar clinical and pathological features, such as some types of muscular dystrophy or systemic diseases, is very important because it implies a different prognosis and response to therapy [1].

## Conclusions

The inclusion of biomarkers such as RIG-I in the routine histopathological analysis of immune-mediated myopathies will help in the definitive diagnosis of DM. Combination of RIG-I and MxA can improve the detection of patients with DM.

## Additional files

Additional file 1: Figure S1. Controls used in the study to assess the specificity of the RIG-I antibody. RIG-I antibody was incubated in non-transfected HEK293 cells (*left*) and in HEK293 cells transfected with the constitutive expression vector pCMV-RIG-I (*right*). Positivity was only observed in those cells transfected with pCMV-RIG-I. Original magnification × 400. (TIF 3652 kb)

Additional file 2: Figure S2. Morphometric study to assess perifascicular atrophy in muscle biopsies stained with anti-MHC-I. Quantification of the fiber size located in the perifascicular and intrafascicle regions in healthy controls (n = 4), and patients with DM (n = 4), PM (n = 4) and MCTD (n = 4). **A** Representative images measuring the Feret’s diameter in a healthy control (*left*) and in a DM (*right*). **B** The diameter of the perifascicular fibers are significantly decreased in DM compared to the other groups while the size of intrafascicle fibers are not significantly different. Original magnification × 40 (*upper pictures*) and digital zoom (*lower pictures*). (TIF 16270 kb)

Additional file 3: Figure S3. Cytoplasmatic expression of MxA in a muscle biopsy of a patient with DM is more prominent in perifascicular fibers. Original magnification × 100. (TIF 9388 kb)
