# Supplementary figures and images for: RIG-I expression in perifascicular myofibers is a reliable biomarker of dermatomyositis
Source: Arthritis Res Ther. 2017 Jul 24;19:174. doi: 10.1186/s13075-017-1383-0 (PMC5525343; doi:10.1186/s13075-017-1383-0)

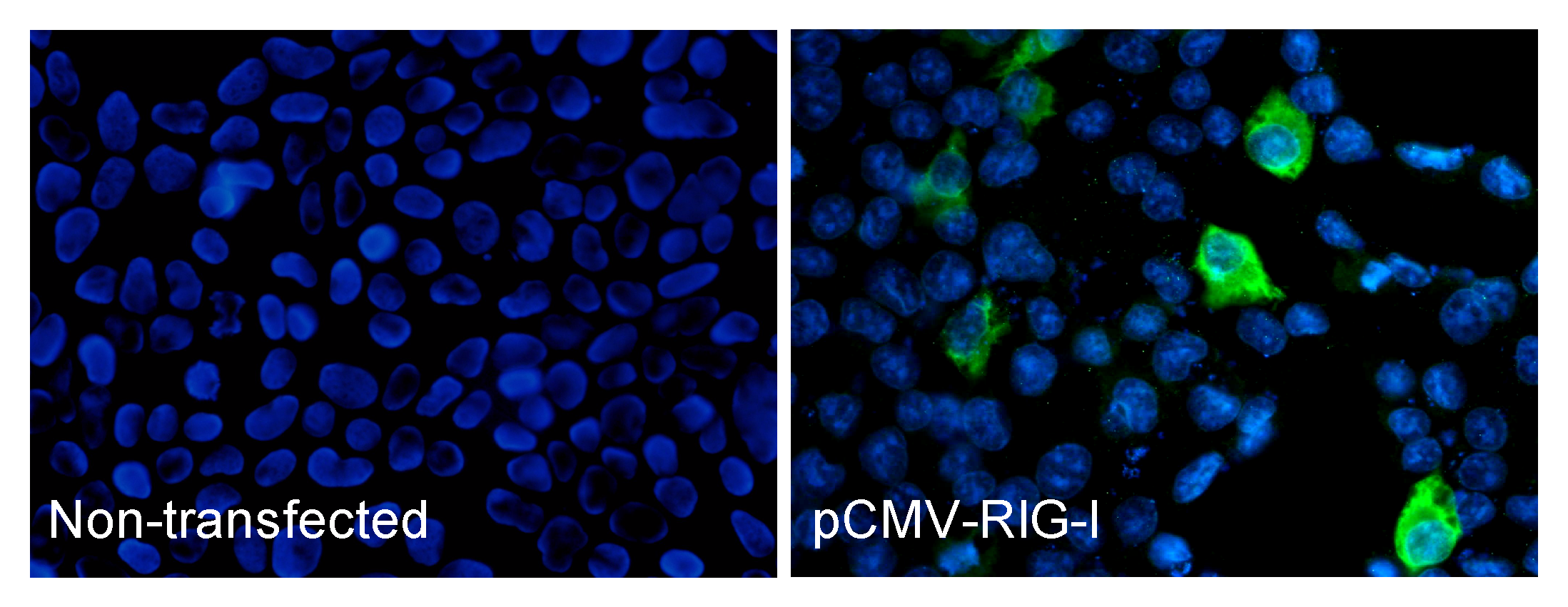

Supplement: Supplementary file 1 — Controls used in the study to assess the specificity of the RIG-I antibody. RIG-I antibody was incubated in non-transfected HEK293 cells (left) and in HEK293 cells transfected with the constitutive expression vector pCMV-RIG-I (right). Positivity was only observed in those cells transfected with pCMV-RIG-I. Original magnification × 400. (TIF 3652 kb) [file 13075_2017_1383_MOESM1_ESM.tif]

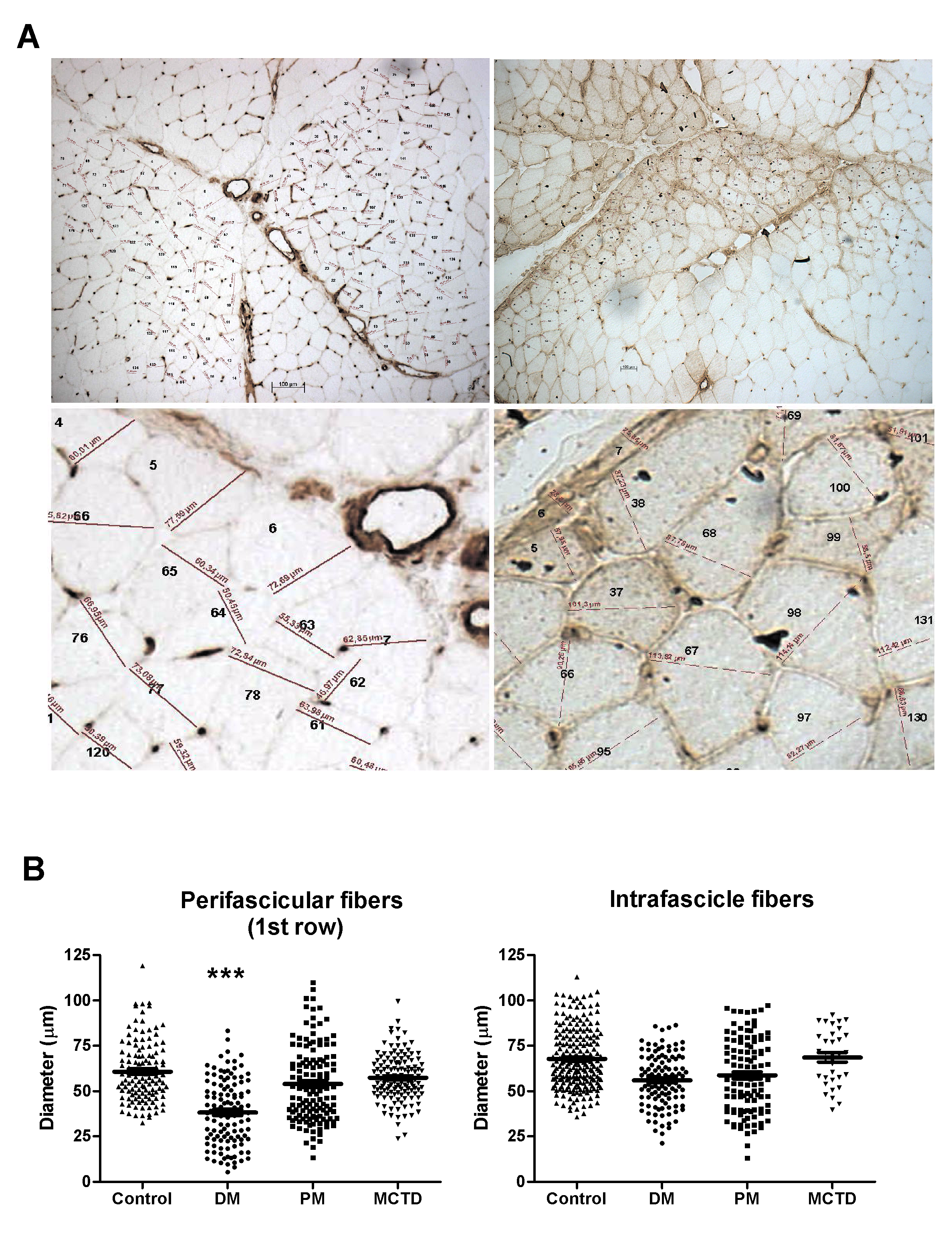

Supplement: Supplementary file 2 — Morphometric study to assess perifascicular atrophy in muscle biopsies stained with anti-MHC-I. Quantification of the fiber size located in the perifascicular and intrafascicle regions in healthy controls (n = 4), and patients with DM (n = 4), PM (n = 4) and MCTD (n = 4). A Representative images measuring the Feret’s diameter in a healthy control (left) and in a DM (right). B The diameter of the perifascicular fibers are significantly decreased in DM compared to the other groups while the size of intrafascicle fibers are not significantly different. Original magnification × 40 (upper pictures) and digital zoom (lower pictures). (TIF 16270 kb) [file 13075_2017_1383_MOESM2_ESM.tif]

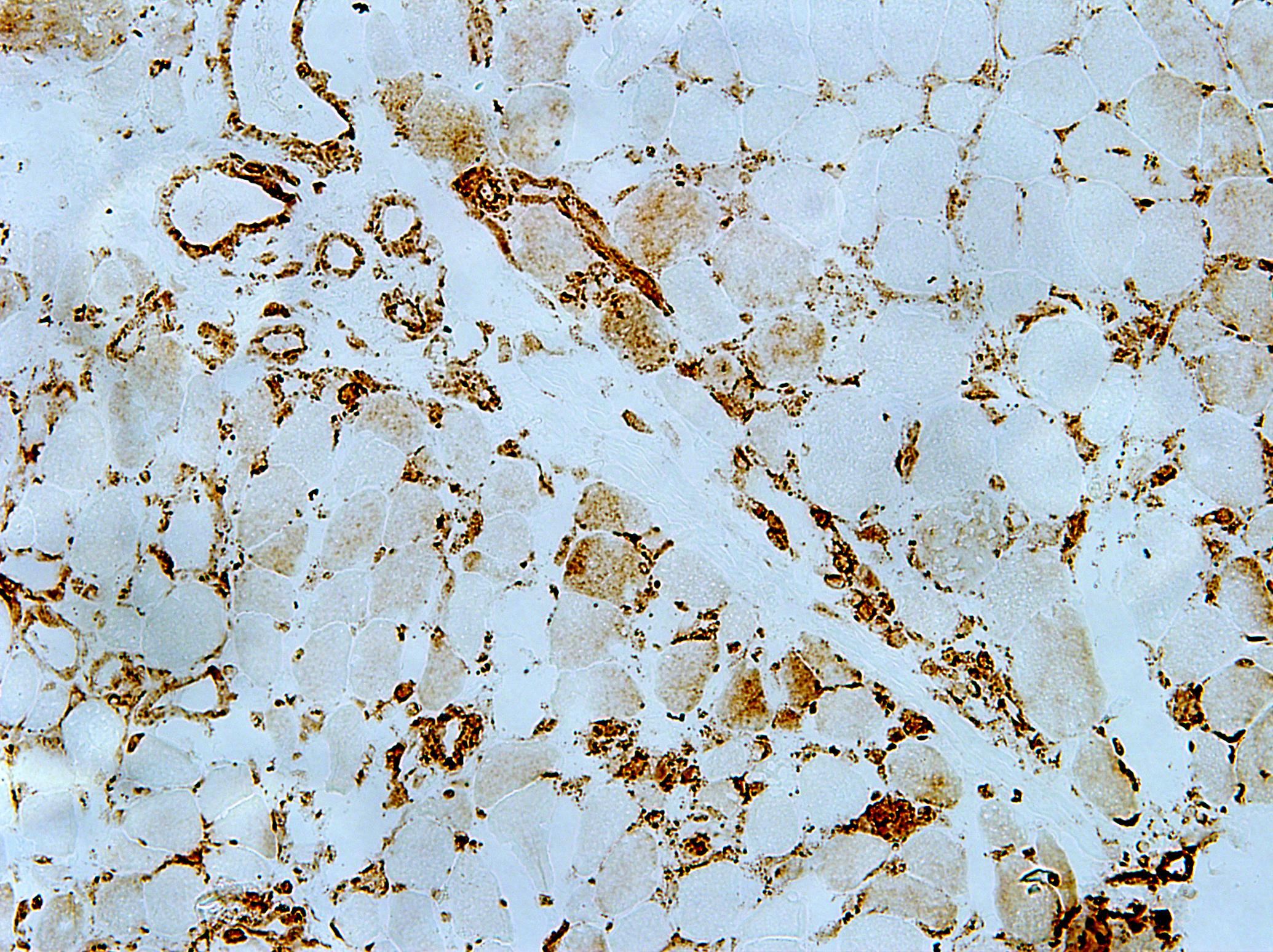

Supplement: Supplementary file 3 — Cytoplasmatic expression of MxA in a muscle biopsy of a patient with DM is more prominent in perifascicular fibers. Original magnification × 100. (TIF 9388 kb) [file 13075_2017_1383_MOESM3_ESM.tif]
